# Systems Pharmacology Study of the Anti-Liver Injury Mechanism of Citri Reticulatae Pericarpium

**DOI:** 10.3389/fphar.2021.618846

**Published:** 2021-04-12

**Authors:** Jianxiong Wu, Xietao Ye, Songhong Yang, Huan Yu, Lingyun Zhong, Qianfeng Gong

**Affiliations:** School of Pharmacy, Jiangxi University of Traditional Chinese Medicine, Nanchang, China

**Keywords:** systems pharmacology, experiment verification, liver injury, mechanism of action, citri reticulatae pericarpium

## Abstract

Liver diseases are mostly triggered by oxidative stress and inflammation, leading to extracellular matrix overproduction and prone to develop into liver fibrosis, cirrhosis and hepatocellular carcinoma. Liver injury (LI) refers to various pathogenic factors leading to the destruction of stem cells that then affect the liver’s normal function, causing a series of symptoms and abnormal liver function indicators. Citri Reticulatae Pericarpium (CRP) is one of the most commonly used traditional Chinese medicines; it contains flavonoids including hesperidin, nobiletin, and tangeretin. CRP has antibacterial, antioxidant, and antitumor effects that reduce cholesterol, prevent atherosclerosis and decrease LI. Here we analyzed the components of CRP and their targets of action in LI treatment and assessed the relationships between them using a systems pharmacology approach. Twenty-five active ingredients against LI were selected based on ultra-performance liquid chromatography-quadrupole/time-of-flight mass spectrometry results and databases. The drug targets and disease-related targets were predicted. The 117 common targets were used to construct a protein-protein interaction network. We identified 1719 gene ontology items in LI treatment, including 1,525 biological processes, 55 cellular components, and 139 molecular functions. These correlated with 49 Kyoto Encyclopedia of Genes and Genomes pathways. These findings suggest that CRP may counteract LI by affecting apoptotic, inflammatory, and energy metabolism modules. *In vitro* experiments suggested that the mechanism may involve hesperidin and naringenin acting on CASP3, BAX, and BCL2 to affect the apoptosis pathway, attenuating liver fibrosis. Naringenin significantly inhibited AKT1 phosphorylation, which in turn mediated activation of the phosphoinositide 3-kinase-Akt signaling pathways against LI. This study provides a reference for systematically exploring the mechanism of CRP’s anti-LI action and is also expands of the application of systems pharmacology in the study of traditional Chinese medicine.

## Introduction

The pathogenetic events leading to liver injury (LI) include oxidative damage, apoptosis, disordered lipid metabolism, and mitochondrial dysfunction. LI refers to one or more results of organ damage, inflammation, bleeding, infection, or other symptoms. Alcohol use is a major predisposing factor for LI (Yang et al., 2020). The annual proportion of alcohol-induced LI has been increasing (Tsutsumi et al., 1994; Vatsalya et al., 2016). Excessive alcohol consumption often causes fatty acid imbalances that trigger liver inflammation.

Traditional Chinese medicine (TCM) is used to treat various diseases. Numerous studies have demonstrated that flavonoids and alkaloids components in TCM prevent or treat LI (Liu, 2016; Zhang et al., 2018). Citri Reticulatae Pericarpium (CRP, orange peel) is a dried and mature pericarp Citrus × aurantium L (Citrus × reticulata Blanco; Rutaceae) (Prc, 2015). CRP extract has excellent biological activity, and the citrus-rich extract is rich in polymethoxy flavonoids that regulate metabolism. CRP extract has excellent biological activity, and citrus-rich extracts rich in polymethoxy flavonoids reduce malnutrition (Zeng et al., 2020). CRP is used in TCM to treat nausea, vomiting, and anemia (Yu et al., 2018). Volatile oils and flavonoids in CRP are considered major components; these include narirutin, nobiletin, and hesperidin (Cui et al., 2019). These components act alone or in combination to combat inflammatory responses and lipid peroxidation, in turn attenuating oxidative stress-induced hepatocyte damage (Lin et al., 2019).

According to the TCM theory, LI is caused by damp heat; viruses invade the liver and lodge in the blood, resulting in liver loss and catharsis regulation. The spleen loses its health and transport capabilities, causing disordered *qi* and blood. CRP is bitter and pungent in taste, warm in nature; it regulates *qi* and dampness, eliminating phlegm and cough, stopping vomiting, eliminating food from the stomach, invigorating the spleen, soothing the liver, and promoting bile discharge (Prc, 2015). A typical TCM formula for treating liver disease is “Chaihu Shugan Powder,” one of CRP’s main components (Nie et al., 2020). TCM treatment reflects three points: overall, dynamic, and syndrome differentiation. Medications regulate bodily functions and balance them by eliminating pathogenic factors and strengthening vital energy.

**FIGURE 1:**
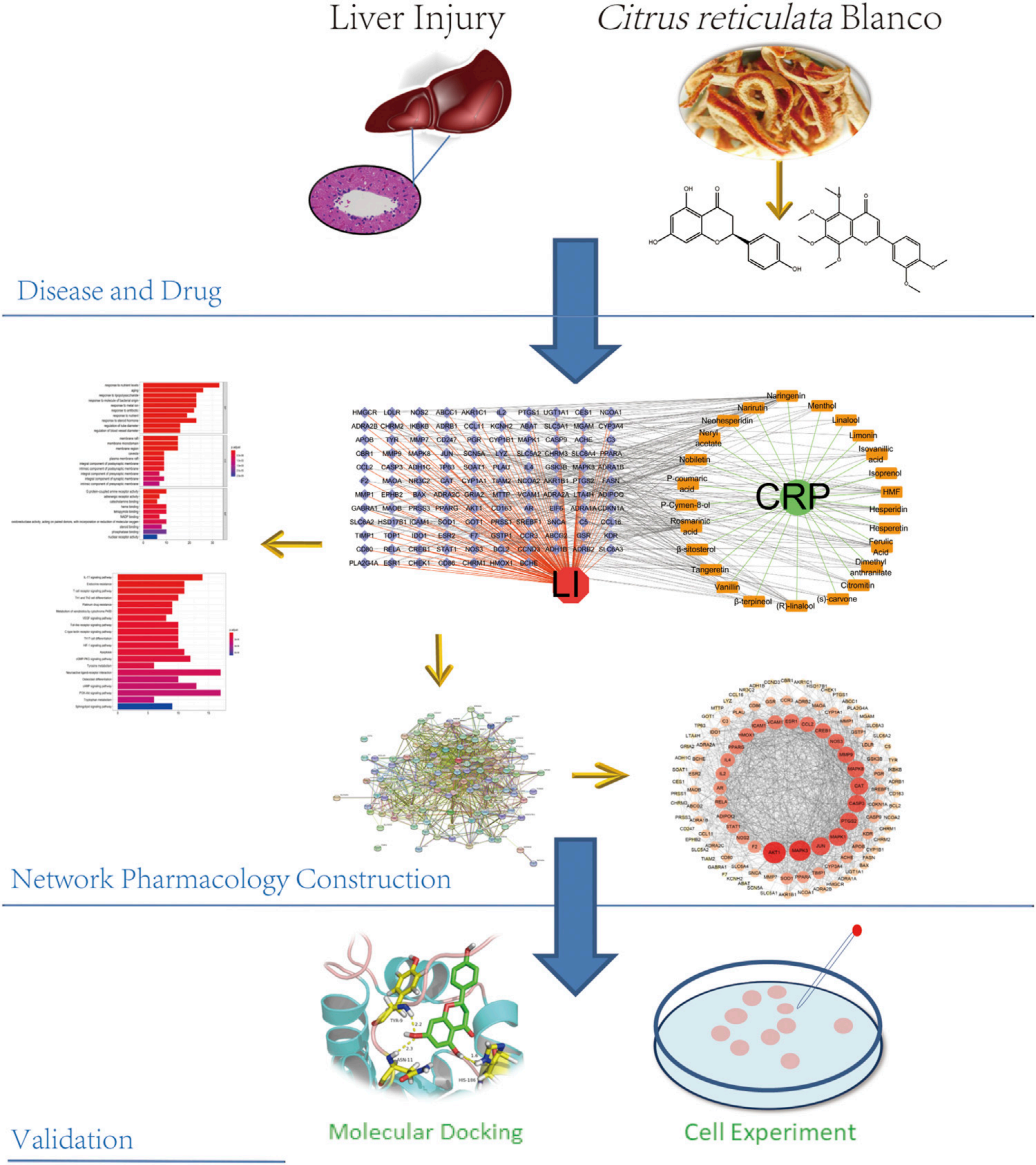
A comprehensive strategy diagram for the study of the mechanism of CRP action on LI.

Systems pharmacology is based on the theory of systems biology, integrating multi-disciplinary technologies such as multi-directional pharmacology, bioinformatics, and computer science to construct multi-level “disease-gene-target-drug” networks. This study analyzed and elucidated the links between CRP and LI using a modern systems pharmacology approach and *in vitro* experiments (Figure 1).

## Materials and Methods

### Instruments and Herb Materials

While collecting and analyzing compounds, we used several instruments, including an ultra-pure liquid chromatograph (UPLC; Nexera X2 LC-30A, Shimadzu Corp. Kyoto, Japan)-hybrid triple-quadruple time-of-flight mass spectrometer (Triple TOF™5600+, AB Sciex, Toronto, Ontario, Canada) with an electrospray ionization source (ESI) and KQ-500 E ultrasonic cleaner (power 500 W, frequency 40 kHz Kunshan Ultrasonic Instrument Co., Ltd. Kunshan, China). The UPLC Titank C18 column (2.1 × 100 mm × 1.8 µm) was purchased from FLM Scientific Instruments Co., Ltd. (Guangzhou, China). Methanol, acetonitrile, and formic acid were purchased from ACS (Washington D.C. United States). Experimental water was ultrapure, obtained using a Milli-QB system (Bedford, MA, United States).

We obtained CRP medicinal materials (batch no. A162002–2) from the Jiangxi Xingan CRP planting base. They were formally identified by Professor Gong Qianfeng of Jiangxi University of TCM as dried and mature pericarps of Rutaceae plant oranges. The remaining samples were stored in the laboratory of the Jiangxi University of TCM. We used 22 standards (purity ≥98%) as references and in subsequent validation studies. Hesperidin (batch no. CHB18023), hesperetin (batch no. CHB180524), nobiletin (batch no. CHB180529), 3′-demethylnobiletin (batch no. CHB180131), 5-*o*-demethylnobiletin (batch no. CHB180321), narirutin (batch no. CHB:1809917), naringenin (batch no. CHB180914), sinensetin (batch no. CHB180126), isosinensetin (batch no. CHB180127), tangeretin (batch no. CHB190125), 5-hydroxy-4′,6,7,8-tetramethoxyflavon (batch no. CHB181119), 3,3′,4′,5,6,7,8-heptamethoxyflavone (batch no. CHB180120), 5,6,7,4′-tetramethoxyflavone (batch no. CHB190106), 5,7,4′-trimethoxyflavone (batch no. CHB181127), diosimin (batch no. CHB180125), diosmetin (batch no. CHB180312), poncirin (batch no. CHB180625), limonin (batch no. CHB180124), nomilin (batch no. CHB180315), naringenin chalcone (batch no. CHB180913), auraptene (batch no. CHB180529), and naringenin-7-O-β-D-glucoside (batch no. CHB181105) were all purchased from Chengdu Chroma-Biotechnology Co., Ltd. (Sichuan, China).

### Screening of Active Compounds

#### Compounds Collected From UPLC-Q-TOF-MS/MS Analysis

Identification technology of compounds and metabolites in CRP has developed rapidly, and mass spectrometry identification is a common technical method (Zheng et al., 2013; Zeng et al., 2017; Li et al., 2019a). We added an appropriate amount of methanol to prepare standard solutions of 50–100 μg/ml and accurately weighed CRP powder (1.0 g), placed it in a conical flask containing 80 ml methanol, mixed it well, treated it with an ultrasound machine for 30 min, passed the solution through filter paper, then increased the volume to 100 ml, and finally filtered the solution through a 0.22 μm microporous membrane to obtain the CRP extraction solution. Ultra-Performance Liquid Chromatography-Quadrupole-Time-of-Flight Tandem Mass Spectrometry (UPLC-Q-TOF-MS/MS) conditions were as follows: chromatographic separation was performed at a flow rate of 0.25 ml/min at 40°C, and elution was performed with a linear gradient program according to various periods. The mobile phase system included solvent A (100% acetonitrile, v/v) and solvent B (0.01% formic acid in water, v/v). The process was solvent A (5–12%) for 5 min, (12–21%) for 10 min, (21–35%) for 10 min, (35–45%) for 11 min, (45–48%) for 4 min, (48–60%) for 1 min, (60–85%) for 9 min, (85–95%) for 1 min, (95%) for 2 min, (95–5%) for 1 min, and isocratic elution at 5% for 2.9 min. The ion source gases 1 and 2 were both set to 50 psi, curtain gas was set to 40 psi, ion spray voltage floating was set to 5500 V in the positive mode and 4500 V in the negative mode, ion source temperature was 500°C, collision energy was 40 V, collision energy spread was 15 V, declustering potential was 100 V, and nitrogen was used as a nebulizer and auxiliary gas. Samples were analyzed in both positive and negative ionization modes with a scanning mass-to-charge (m/z) range from 50 to 1,000. Data were collected in information-dependent acquisition mode and were analyzed using the Analyst TF 1.6 data acquisition workstation and PeakView®1.2 software. We compared the fragment ion information obtained for various compounds with that of the 22 standards and identified several CRP compounds. Also, we studied and analyzed the literature documenting fragment ion information of CRP components in detail carefully, matched it with our information of multiple compounds, and identified additional compounds.

#### Compounds Collected From Databases

To collect drug ingredients more thoroughly, we used The Encyclopedia of Traditional Chinese Medicine (http://www.tcmip.cn/ETCM), the Traditional Chinese Medicine Systems Pharmacology database and Analysis Platform (TCMSP, http://lsp.nwu.edu.cn/tcmsp.php), and the TCM database Taiwan (http://tcm.cmu.edu.tw). All three online databases were searched for compounds, using the Chinese name “ChenPi” and the Latin name *Citrus reticulata* Blanco.

#### Compound Identification Analysis

We integrated the collected compounds and established a CRP chemical composition. First, the concept of oral bioavailability (OB) was introduced. OB refers to the percentage of unmodified drug entering the circulatory system after oral administration, representing drug utilization efficiency. Higher OB correlates with a higher possibility of clinical usefulness (Lv W. et al., 2020). Drug-likeness (DL) refers to the potential of a compound to become a drug (Huang et al., 2017). Molecules with OB ≥ 30% or DL ≥ 0.18 are considered to have better pharmacological effects (Zhang M. et al., 2020). We referred to this principle to select active ingredients for the subsequent step of the analysis. Considering the special biological activities of some compounds, we included them in the final candidate component database.

### Identification of the Related Targets and Gene Symbols of CRP Compounds

We used information in the TCMSP (http://lsp.nwu.edu.cn/tcmsp.php) and Swiss Target prediction (STP, https://www.swisstargetprediction.ch/) to extract protein targets for each CRP component (Mu et al., 2020). After removing redundant information, the targets that could interact with each CRP component were retained.

### Acquisition of Liver Injury Gene Targets

The gene targets for LI was obtained from the GeneCards database (https://www.genecards.org/, version 4.9.0) and the Online Mendelian Inheritance in Man database (OMIM, http://www.omim.org/, updated on February 28, 2019) (Mu et al., 2020; Xu et al., 2020). We performed keyword searches using “liver injury” and carefully screened the results. Finally, we obtained genes associated with LI-related diseases.

### Drug-Compounds-Genes-Disease Network Construction

To obtain overlapping targets, we first cross-contrasted CRP-related targets with LI-related targets. We then built a complex information network (D-C-G-D) based on the interactions among drugs (CRP), compounds, gene symbols, and disease (LI) using Cytoscape software (version 3.8.0) and drew a schematic diagram.

### Protein-Protein Interaction Network Construction

PPI data were obtained from the STRING database (https://string-db.org/, Version 11.0, updated on January 19, 2019). The interaction relationships provided by STRING are based on the confidence score and can be used to filter and assess functional genomics data (Tao et al., 2020). The organism species was set to *Homo sapiens* (Human), and the merged gene symbols were analyzed. We also used the BioGPS database (https://biogps.org) to identify proteins’ higher expression in some major organs (Gu et al., 2020). The heat map was generated using Heml software (version 1.0).

### Gene Ontology and Kyoto Encyclopedia of Genes and Genomes Pathway Enrichment

Gene Ontology (GO) and Kyoto Encyclopedia of Genes and Genomes (KEGG) enrichment analyses were performed using R software (version 4.0.2). We used the R package “BiocGenerics,” a classic and practical tool specially designed for biologists to conduct an in-depth analysis of these targets (Yu et al., 2012). All enriched entries were determined using the Bioconductor database (http://bioconductor.org/).

### Computational Validation of Ingredients-Targets Interactions

Molecular docking is a drug design method that considers receptors’ characteristics and the mode of interaction between receptors and drug molecules (Yang et al., 2019). We docked a variety of active components and key targets (BAX, BCL2, and caspase-3 [CASP3]) for a total of six component-target interactions. We obtained data from the RCSB Protein Data Bank (PDB, www.rcsb.org); the X-ray crystal structures of the key targets BAX, BCL2, and CASP3 were obtained and confirmed concerning the relevant literature; their PDB IDs were 1F16, 1G5M, and 5IBC, respectively. After processing targets and compounds using PyMOL software (version 1.3), docking work was performed using AUTODOCK VINA software (Version 1.1.2, Scripps Research, San Diego, CA, United States). The required input files for the AutoDock program were prepared using AutoDock tools.

### Experimental Validation

#### Cell Culture

The LX-2 cell line was purchased from Beina Chuanglian Biotechnology Research Institute (HeNan, China). Cells were cultured in high-glucose Dulbecco’s minimum essential medium (Solarbio, Beijing, China) containing 10% fetal bovine serum (Tianhang Biotechnology, Zhejiang, China) in a 37°C, 5% CO_2_ incubator. Dimethyl sulfoxide was used to dissolve treatment compounds.

#### CCK-8 Assay for Cell Viability

Logarithmic growth phase cells were plated in 96-well plates (5 × 10^3^ cells per well) and cultured overnight in a 37°C, 5% CO_2_ incubator. The following day, various hesperidin and naringenin (0, 25, 50, 75, 100, and 125 μM) were added to fresh medium. After 12 h, the model and administration groups were incubated with 5 ng/ml transforming growth factor (TGF)-β1 for 24 h, and then 10 μL CCK-8 assay solution (Solarbio) was added to each well. A microplate reader (Nanjing Detie Experimental Equipment Co., Ltd. Nanjing, China) was used to measure the optical density (OD) value around 450 nm. Cell survival was calculated as: $\frac{Ab}{AC}\times 100\%$ (Ab and Ac stand for absorbance and absorbance of the control, respectively).

#### Real-Time Quantitative Polymerase Chain Reaction (qRT-PCR)

Total RNA was extracted using TRIzol^®^ Reagent (Thermo Scientific, Waltham, MA, USA) and was reverse-transcribed with oligo-DT using HiScript™ Reverse transcriptase (Thermo Scientific) according to manufacturer instructions. The primers used were synthesized by Tsingke (Beijing, China). The sequences (forward and reverse, respectively), were as follows:

5′-CAT​GGG​CTG​GAC​ATT​GGA​CT-3′ and 5′-AAA​GTA​GGA​GAG​GAG​GCC​GT-3′ for BAX; 5′-TGA​GTG​CTC​GCA​GCT​CAT​AC-3′ and 5′-TTC​CCT​GAG​GTT​TGC​TGC​AT-3′ for CASP3; 5′-CTT​TGA​GTT​CGG​TGG​GGT​CA-3′ and 5′-GAA​ATC​AAA​CAG​AGG​CCG​CA-3′ for BCL2; and 5′-GAA​AGC​CTG​CCG​GTG​ACT​AA-3′ and 5′-TTC​CCG​TTC​TCA​GCC​TTG​AC-3′ for the internal control glyceraldehyde 3-phosphate dehydrogenase (GAPDH). The GenBank accession numbers of BAX, CASP3, BCL2, and GAPDH used were NM_001291428.2, NM_001354777.2, NM_000633.3, and NM_001256799.3, respectively. Reverse transcription reaction conditions were: 30°C 10 min, 42°C 60 min, 99°C 5 min, and 4°C 5 min. The synthesized cDNA was stored at −20°C for later use. Gene-expression data were normalized to that of the endogenous control GAPDH. The 2-^△△^ Ct method was used as the basis for relative gene expression.

#### Western Blot

LX-2 cells were collected, lyzed in radioimmunoprecipitation assay buffer, and then centrifuged at 13,000 rpm for 10 min at 4°C. Bicinchoninic acid protein analysis kits (Solarbio) were used to calculate the samples’ protein concentrations based on standard curves. Protein samples (30 μg/sample) were added to the loading buffer, heated at 97°C for 6 min, and centrifuged at room temperature. After loading the samples, they were separated on 4% sodium dodecyl sulfate-polyacrylamide gels with voltage 150 V. The proteins were transferred to polyvinylidene membranes (Millipore, MA, United States) using a electroporation device. Next, the membranes were blocked with 5% skimmed milk powder in phosphate-buffered saline for 30 min. After incubation with a single antibody, a ChemiScope Mini 3,300 chemiluminescence imaging system (Shanghai, China) was used to image the blots. The primary antibodies were from Dingguo Changsheng Biotechnology Co., Ltd. (Beijing, China). Band intensity was analyzed using Quantity One software (version 4.6.6).

### Statistical Analyses

The significance of results was determined based on one-way analysis of variance using Prism 8.0.1 software (Graph Pad Inc. San Diego, CA, United States). All experiments were performed in triplicate, and data are presented as mean ± SD. Differences were considered significant at *p* < 0.05.

## Results

### Screening of Active Compounds Collected From UPLC-Q-TOF-MS/MS Analysis

**FIGURE 2:**
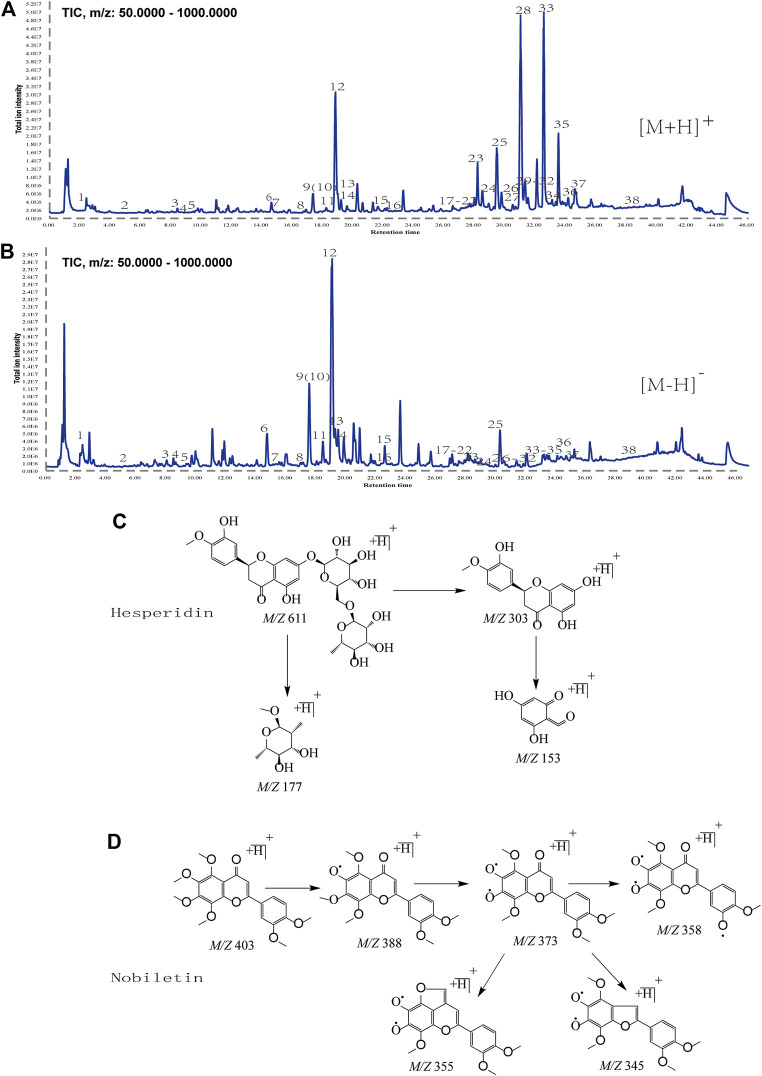
**(A, B)** Total ion chromatograms of CRP obtained by UPLC/Q-TOF-MS/MS in positive and negative ion mode. **(C, D)** Fragmentation Law of hesperidin and nobiletin by Mass Spectrometry.

We showed a typical total ion chromatogram of alcohol-soluble components extracted from CRP (Figures 2A,B) and the mass spectrometric cleavage law of hesperidin and nobiletin (Figures 2C,D). By sampling the primary mass spectrum, we determined each component’s relative molecular weight and then obtained fragmentation information based on the secondary mass spectrum. Based on the retention time and mass spectrometric information of the standard material and the compounds reported in the literature, we identified a total of 38 compounds. Twenty-two of these (8, 9, 11, 12, 14, 16, 17, 18, 19, 21, 22, 24, 25, 26, 27, 28, 29, 31, 32, 34, 36, and 37) were identified through careful comparison of information between the samples and standard pure substances. The details are shown in Supplementary Table S1. Through the comparison of the multi-level fragment ion information recorded in the relevant literature and the analysis of the fragmentation law, the other 16 components were identified. Table 1 presents the details of the 38 compounds.

**TABLE 1:** CRP compounds identified by UPLC/Q-TOF-MS/MS.

No	Molecule name	Molecular formula	ESI-MS	Error (ppm)	tR/min	ESI-MS/MS	References
1	Synephrine	C_9_H_13_NO_2_ [M + H]^+^	168.1018	−0.8	2.24	135.0679, 134.0601, 107.0498, 91.0554, 77.0405	Zheng et al. (2019)
2	Vanillic acid	C_8_H_8_O_4_ [M-H]^-^	167.03606	6.5	5.47	167.0302, 152.0135, 122.9371, 108.0229, 78.9459	Zheng et al. (2019)
3	Scopoletin	C_10_H_8_O_4_ [M + H]^+^	193.04982	1.5	8.35	193.0513, 178.0263, 150.0322, 133.0298, 122.0370	Zheng et al. (2020a)
4	Caffeic acid	C_9_H_8_O_4_ [M-H]^-^	179.03578	4.5	9.5	135.0469, 134.0400, 91.0000, 89.0556, 89.0438	Zheng et al. (2019)
5	Lucenin-2	C_27_H_30_O_16_ [M + H]^+^	611.1632	4.2	9.62	473.1115, 425.0908, 395.0796, 353.0682, 341.0691	Zheng et al. (2019)
6	Ferulic acid	C_10_H_10_O_4_ [M-H]^-^	193.05114	2.6	14.77	178.0267, 134.0387, 133.0308, 132.0217	Zheng et al. (2019)
7	Apigenin‐8‐C‐glucoside	C_21_H_20_O_10_ [M + H]^+^	433.11435	3.3	15.18	433.1143, 415.1045, 397.0952, 313.0727, 283.0619	Zheng et al. (2019)
8	Diosmetin‐6‐C‐glucoside	C_22_H_22_O_11_ [M + H]^+^	463.12507	3.4	16.75	445.1168, 427.1058, 367.0847, 343.0845, 313.0734	Zheng et al. (2019)
9	Narirutin	C_27_H_32_O_14_ [M + H]^+^	581.18783	2.3	17.35	273.3773, 273.0777, 195.0302, 153.0193, 85.0307	*
10	Naringenin-7-O-β-D-glucoside	C_21_H_22_O_10_ [M + H]^+^	435.12943	2	17.36	273.0777, 153.0195, 147.0452	*
11	Homoeriodictyol	C_16_H_14_O_6_ [M + H]^+^	303.08759	4.2	18.82	177.0560, 153.0195, 149.0611, 145.0299, 117.0351	Zheng et al. (2019)
12	Hesperidin	C_28_H_34_O_15_ [M + H]^+^	611.19883	2.9	18.83	303.0867, 177.0542, 153.0183	*
13	Diosimin	C_28_H_32_O_15_ [M + H]^+^	609.18374	3.8	19.2	302.2107, 301.9498, 301.7986, 301.5234, 301.0729	*
14	Scoparone	C_11_H_10_O_4_ [M + H]^+^	207.06564	2.2	19.59	207.0671, 191.0353, 163.0404, 151.0769, 107.0507	Zheng et al. (2020b)
15	Poncirin	C_28_H_34_O_14_ [M + H]^+^	595.20393	3	23.29	287.0929, 263.0568, 195.0303, 153.0191, 85.0305	*
16	Isosakuranetin	C_16_H_14_O_5_ [M + H]^+^	287.09258	4.1	23.3	153.0198, 161.0609, 133.0659, 287.0930	Zheng et al. (2019)
17	Naringenin	C_15_H_12_O_5_ [M + H]^+^	273.07576	0	26.47	153.0181, 147.0445, 119.0491, 91.0556	*
18	Naringenin chalcone	C_15_H_12_O_5_ [M + H]^+^	273.07632	2.1	26.52	153.0198, 147.0453, 119.0504, 91.0564	*
19	Diosmetin	C_16_H_12_O_6_ [M + H]^+^	301.07162	3.2	26.84	301.0740, 286.0501, 195.0457, 168.0066, 160.0530	*
20	Hesperetin	C_16_H_14_O_6_ [M + H]^+^	303.08759	4.2	27.38	177.0556, 153.0195, 145.0294, 117.0347, 303.0884	*
21	Jaceosidin	C_17_H_14_O_7_ [M + H]^+^	331.08241	3.6	27.8	331.0845, 316.0599, 301.0361, 273.0411, 168.0062	Zheng et al. (2020b)
22	Isosinensetin	C_20_H_20_O_7_ [M + H]^+^	373.12968	4	28.19	357.0974, 343.5625, 343.0819, 315.0865	*
23	3′-demethylnobiletin	C_20_H_20_O_8_ [M + H]^+^	389.12468	4.1	28.5	359.0765, 344.0532, 313.0713	*
24	6-Demethoxytangeretin	C_19_H_18_O_6_ [M + H]^+^	343.11925	4.8	29.44	313.0787, 285.0755, 181.0129, 153.0180, 313.5300	Zheng et al. (2020a)
25	Sinensetin	C_20_H_20_O_7_ [M + H]^+^	373.12979	4.3	29.5	357.0969, 343.0812, 339.0864, 329.1023, 312.0992	*
26	Limonin	C_26_H_30_O_8_ [M + H]^+^	471.20333	4.2	29.77	425.1981, 213.0922, 161.0605, 95.0146	*
27	5,7,4′-trimethoxyflavone	C_18_H_16_O_5_ [M + H]^+^	313.10816	3.6	30.51	298.0857, 297.0778, 270.0904, 269.0828	*
28	Nobiletin	C_21_H_22_O_8_ [M + H]^+^	403.13999	3.1	31.03	388.1125,373.0911, 358.0683, 355.0810, 345.0950	*
29	5,6,7,4′-tetramethoxyflavone	C_19_H_18_O_6_ [M + H]^+^	343.11934	5	31.32	327.0867, 313.0709, 299.0916, 282.0885	*
30	Nomilin	C_28_H_34_O_9_ [M + H]^+^	515.22891	2.6	31.51	515.2302, 469.2238, 205.0508, 187.0767, 161.0612	*
31	Chrysosplenetin B	C_19_H_18_O_8_ [M + H]^+^	375.10916	4.6	31.52	375.1096, 360.0862, 345.0623, 327.0518, 197.0095	Zheng et al. (2020b)
32	3,3′,4′,5,6,7,8-heptamethoxyflavone	C_22_H_24_O_9_ [M + H]^+^	433.15146	5	32.1	417.1202, 403.1035, 385.0935	*
33	Tangeretin	C_20_H_20_O_7_ [M + H]^+^	373.12991	4.6	32.55	344.2913, 343.0820, 328.0587, 325.0717, 297.0761	*
34	Obacunone	C_26_H_30_O_7_ [M + H]^+^	455.20774	-0.7	32.71	455.2080, 409.2024, 175.0760, 161.0601, 133.0643	Zheng et al. (2020a)
35	5-O-Demethylnobiletin	C_20_H_20_O_8_ [M + H]^+^	389.12458	3.8	33.52	373.0917, 359.0751, 341.0643, 197.0072	*
36	Atractylenolide Ⅱ	C_15_H_20_O_2_ [M + H]^+^	233.1542	2.6	34.15	187.1494, 145.1024, 131.0865, 91.0556, 105.0709	Zheng et al. (2020b)
37	5-Hydroxy-4′,6,7,8-tetramethoxyflavone	C_19_H_18_O_7_ [M + H]^+^	359.11422	4.7	34.69	329.0675, 311.0570, 197.0093	*
38	Auraptene	C_19_H_22_O_3_ [M + H]^+^	299.16455	1.3	38.66	283.0539, 267.0223, 250.9903, 163.0404, 119.0488	*

^*^ Identified by comparison with standards.

### Compounds Collected From Databases

We searched online databases commonly used for TCM ingredients, The Encyclopedia of Traditional Chinese Medicine (http://www.tcmip.cn/ETCM), Traditional Chinese Medicine Systems Pharmacology database and Analysis Platform (http://lsp.nwu.edu.cn/tcmsp.php), and the TCM database Taiwan (http://tcm.cmu.edu.tw) to produce a more comprehensive compound list (Yu et al., 2020; Zhu and Hou, 2020). We collected a total of 65 compounds from these databases as candidates for CRP studies (Supplementary Table S2).

### Compound Identification Analysis

We collated the results of previous work, combined the components obtained using UPLC/Q-TOF-MS/MS analysis and all CRP components identified in online databases. After removing repeated items, we established a complete CRP internal compound composition library. To screen out the active ingredients of CRP, the classical absorption, distribution, metabolism, and excretion (ADME) parameters of OB and DL were used to screen them. We found five ingredients that met these conditions; however, this result was far below our expectations. CRP contains a large number of flavonoids, including hesperidin and naringin (Dong et al., 2010). Flavonoid glycosides are more easily absorbed after hydrolysis into flavonoid aglycones (Hostetler et al., 2012), and hesperidin is one of the typical constituents (Nectoux et al., 2019). The OB of hesperidin was 13.33%, which was <30%; however, it is an active compound that is significantly effective against LI (Adefegha et al., 2017; Elhelaly et al., 2019). Narirutin has an OB of 8.15% and a DL of 0.75. However, it alleviates LI by regulating protein phosphorylation in the mitogen-activated protein kinase (MAPK) pathway, which may be related to lipid metabolism (Jianping et al., 2019; Gao et al., 2020). Tangeretin has an OB of 21.38% and a DL of 0.43, but it can ameliorate oxygen-glucose deprivation-induced LI through the JNK signaling pathway (Wu et al., 2019). Rosmarinic acid has an OB of 1.38% and a DL of 0.35; it can activate the AMP-activated protein kinase (AMPK) pathway and inhibit inflammation through nuclear factor changes erythroid 2-related factor and nuclear factor-κB signaling and ameliorates obesity and LI (Gyhye et al., 2019). Another mechanism by which compounds counteract LI is antioxidation, and the candidate *p*-Cymen-8-ol has both antioxidant effects and antibacterial activity (Pires et al., 2006; Santana et al., 2009). Although some components have relatively low kinetic values, they have excellent biological activity; therefore, we still consider them active CRP components. For these reasons, we propose that as long as the candidate components in CRP have good biological activity and intersect with LI’s target, they can be considered active CRP compounds for this indication. We selected 25 components as the final active compounds in the candidate compound library (Table 2).

**TABLE 2:** **|**Final selected compounds per the details of the active compounds in CRP.

NO.	Molecule name	Cas no.	Structure	References
1	β-sitosterol	83–46−5	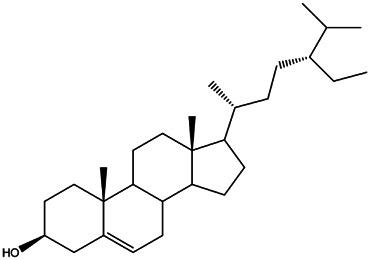	Luo et al. (2019)
2	Naringenin	480–41−1	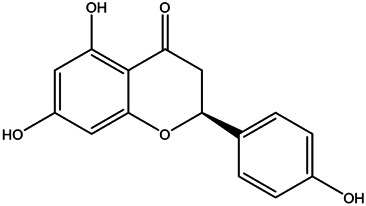	*
3	Hesperetin	520–33−2	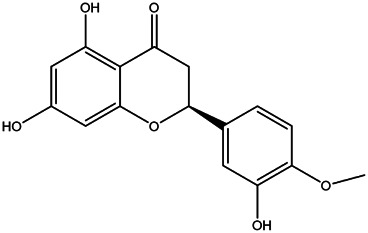	*
4	Citromitin	3570–71−6	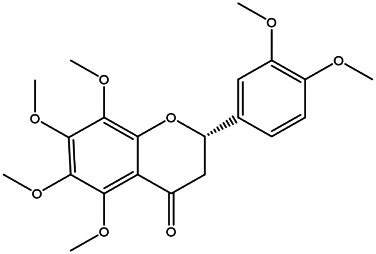	Luo et al., (2019)
5	Nobiletin	478–01−3	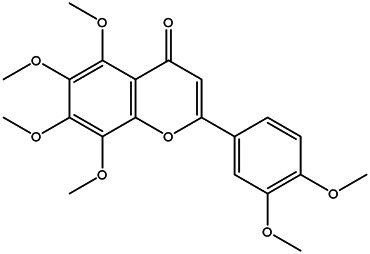	*
6	(R)-linalool	126–91−0	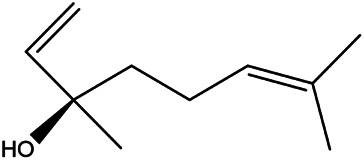	Johnson et al. (2019)
7	Isovanillic acid	645–08−9	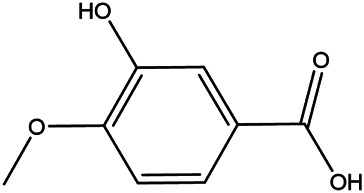	Zheng et al. (2019)
8	P-cymen-8-ol	1,197–01−9	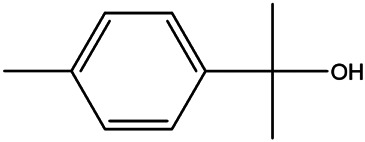	Yi et al. (2015)
9	Dimethyl anthranilate	85–91−6	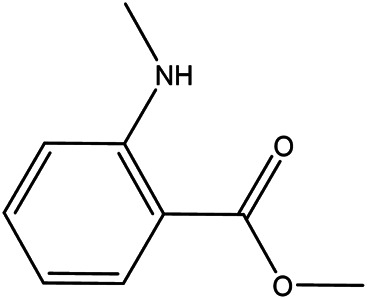	Li et al. (2019b)
10	Isoprenol	763–32−6	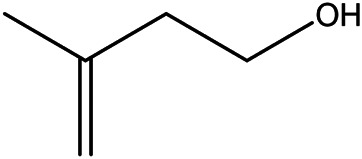	Run-Xia et al. (2013)
11	Neryl acetate	141–12−8	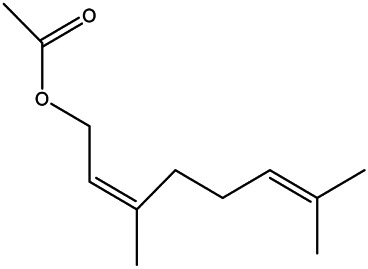	Yi et al. (2015 ^)^
12	Vanillin	121–33−5	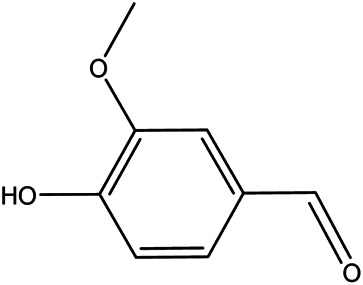	Zheng et al. (2020a)
13	β-terpineol	138–87−4	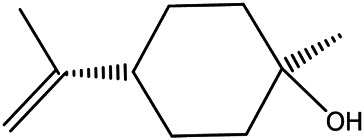	Qiao et al. (2008 ^)^
14	HMF	67–47−0	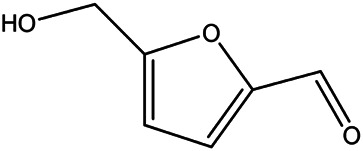	Xiao et al. (2009 ^)^
15	P-coumaric acid	501–98−4	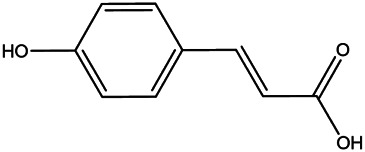	Feumba Dibanda et al. (2020 ^)^
16	Ferulic acid	1,135–24−6	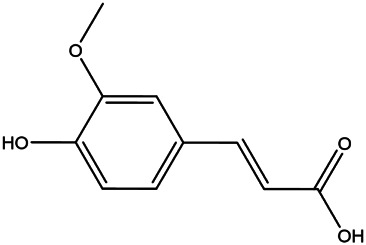	*
17	(S)-carvone	2244–16−8	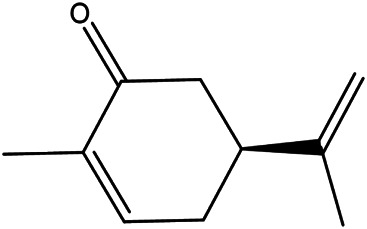	Wang et al. (2008 ^)^
18	Linalool	78–70−6	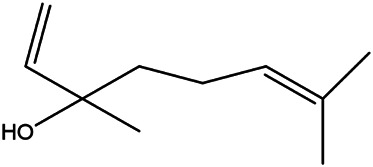	Lv et al. (2020b)
19	Menthol	2216–51−5	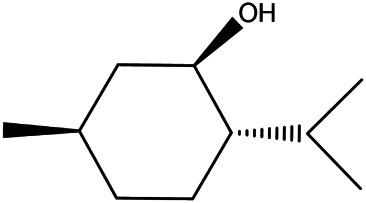	Kim et al. (2018)
20	Hesperidin	520–26−3	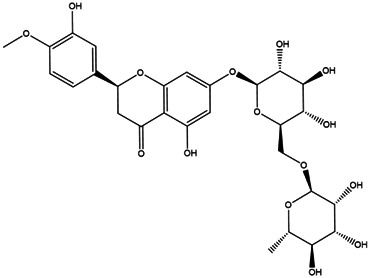	*
21	Tangeretin	481–53−8	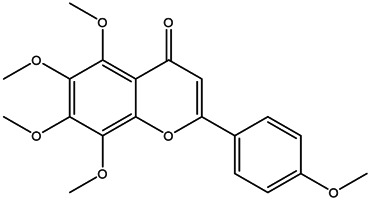	*
22	Rosmarinic acid	20,283–92−5	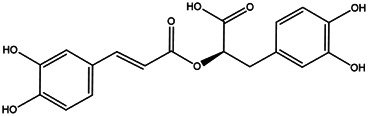	Feumba Dibanda et al. (2020)
23	Neohesperidin	13,241–33−3	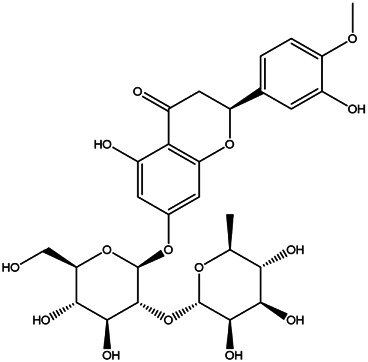	Zheng et al. (2020b)
24	Limonin	1,180–71−8	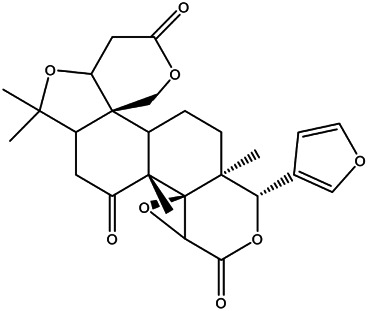	*
25	Narirutin	14,259–46−2	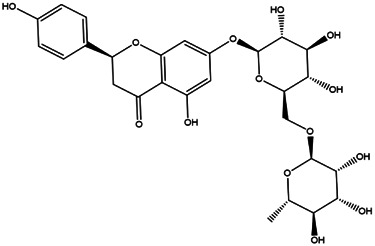	*

^*^ Identified using UPLC/Q-TOF-MS/MS.

### Identification of Related Targets and Gene Symbols of Compounds in CRP

After we collected the relevant proteins from the TCMSP and STP databases, they were converted in the UniProt database, and redundant terms were removed to obtain 25 components in CRP and the 126 known associated target symbols (Supplementary Table S3).

### Acquisition of Known Therapeutic Gene Targets for Liver Injury

A subset of the LI-related targets was obtained from the GeneCards database. We obtained 7372 known LI target symbols. Also, 92 known therapeutic target data for LI were obtained from OMIM. After eliminating redundancies, a total of 7442 known therapeutic targets for LI were collected (Supplementary Table S4).

### Drug-Compounds-Genes-Disease Network Construction

**FIGURE 3:**
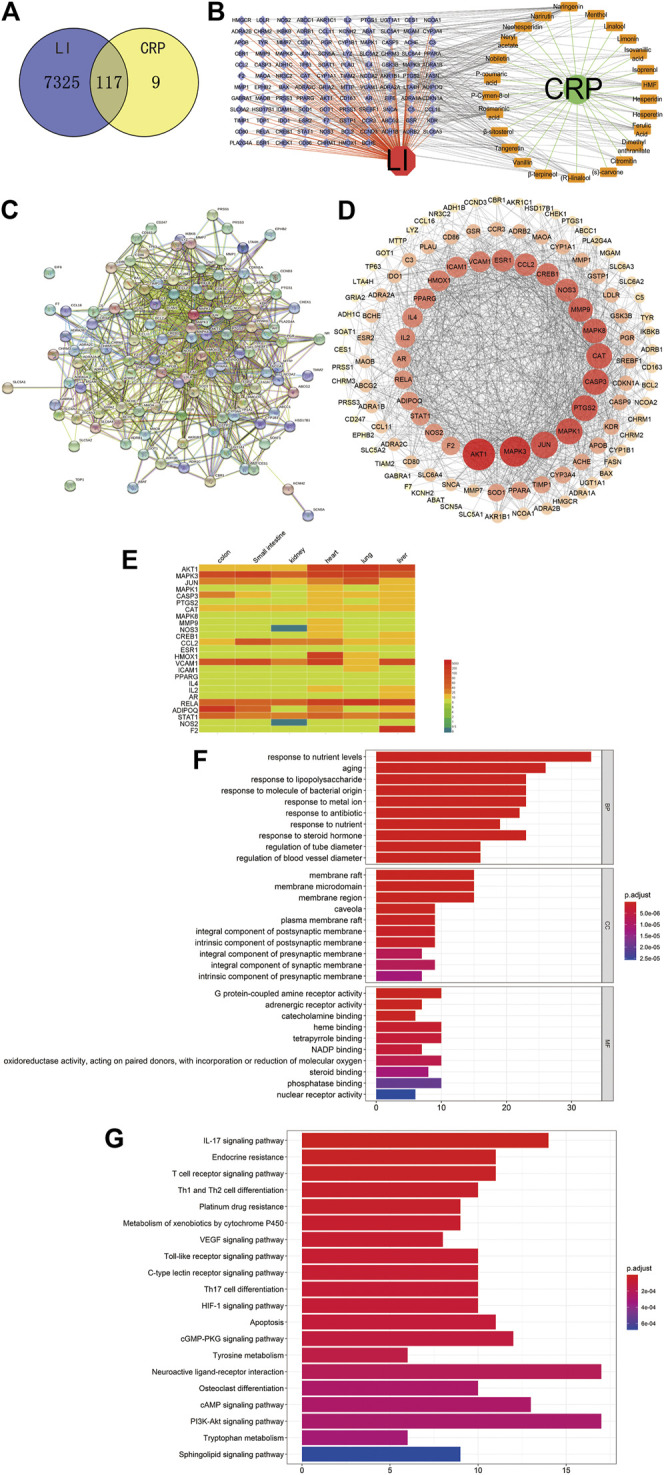
**(A)** Venn diagram of related targets of CRP and LI. **(B)** D-C-G-D network. Green and red nodes indicate CRP and LI, respectively. Twenty-five orange nodes represent active ingredients in CRP; 117 blue nodes represent overlapping gene symbols between diseases and drugs, with edges indicating that nodes can interact; red edges indicate the action of LI with genes, green edges indicate the interaction of CRP with active ingredients, and gray edges indicate the interaction of active ingredients with genes. **(C)** The PPI network was obtained from the STRING database platform. **(D)** The PPI network is arranged according to degree value. **(E)** The expression of the core target in vital organs. Red and blue indicate higher and lower expression, respectively. **(F)** Bar graph of GO function enrichment of overlapping targets. **(G)** Bar graph of KEGG enrichment of overlapping targets. The *Y*-axis represents GO terms or KEGG pathways. The *X*-axis indicates the number of genes enriched in this pathway. The redder the color, the smaller the p. adjust value; it also indicates the reliability and importance. The bluer the color, the greater the p. adjust value.

We contrasted the obtained drug targets with disease-related genes to obtain their common cross genes. We constructed Venn diagrams (Figure 3A) with 117 overlaps of 7442 disease gene symbols and 126 drug-gene symbols. To intuitively demonstrate how CRP counteracts LI, we constructed a D-C-G-D network using Cytoscape software (Figure 3B). D-C-G-D details are in Supplementary Table S5.

### Protein-Protein Interaction Network Construction

An action relationship map between genes was obtained after 117 overlapping genes were entered into the STRING online database (Figure 3C). These genes may be critical targets for LI treatment using CRP. The gene names are listed in Supplementary Table S6. After removing two free genes, we calculated the topological indices DC, BC, and CC for 115 genes. Degree (DC) indicates how much a gene is linked to other genes, suggesting that the gene may be necessary for the process of CRP counteracting LI. Detailed information about genes and topological indices is provided in Supplementary Table S7. We calculated the mean value of the 115 overlapping gene degrees to be 29.42. All genes with degree values >29.42 were selected, resulting in 25 core targets for interaction networks with other genes. After processing these associated targets using Cytoscape software, the inner loop of Figure 3D shows 25 genes with higher DC values. The deeper red color and large nodes indicate stronger correlations, and these may be critical genes for the CRP treatment of LI. To investigate LI and other major organs’ connection, we considered these core targets’ expression in various organs. According to the presented results of the heat map (Figure 3E), the 25 core targets’ expression was generally similar in the main organs. For example, AKT1 and MAPK3 were highly expressed in the liver, heart, lung, kidney, small intestine, and colon, while expression levels of PPARG and IL4 were low in these organs. This finding suggests that, in addition to the liver, LI is also closely related to other organs, consistent with the fact that TCM considers the human body as a whole. The specific expression of these core targets in various organs is shown in Supplementary Table S8.

### Gene Ontology and Kyoto Encyclopedia of Genes and Genomes Pathway Enrichment

To better summarize their specific functions, we performed GO enrichment analysis of 117 consensus genes and classified and elaborated them according to three modules: biological process (BP), cellular component (CC), and molecular function (MF), including 1525 GO terms for BP terms, 55 GO terms for CC terms, and 139 GO terms for MF terms, for a total of 1719 enriched GO terms. Based on the p. adjust value (*p* < 0.01), we selected the first ten terms from small to large for the enrichment results of BP, CC, and MF (Figure 3F). The BP analysis results revealed essential ways in which genes participate in bodily processes. These processes involve responses to nutrient levels (GO: 0031667), aging (GO: 0007568), responses to lipopolysaccharide (GO: 0032496), responses to a molecule of bacterial origin (GO: 0002237), and responses to metal ion (GO: 0010038). CC analysis then identified the sites where these intersection genes function intracellularly and extracellularly, primarily involving membrane raft (GO: 0045121), membrane microdomain (GO: 0098857), membrane region (GO: 0098589), caveola (GO: 0005901), and plasma membrane raft (GO: 0044853). Similarly, from MF analysis, we understand the forms of protein and target binding, primarily involving G protein-coupled amine receptor activity (GO: 0008227), adrenergic receptor activity (GO: 0004935), catecholamine binding (GO: 1901338), heme-binding (GO: 0020037), and tetrapyrrole binding (GO: 0046906). Overall, GO enrichment analysis results suggest that most of the effects of CRP on the liver occur in cell proliferation, regulation of enzyme activity, and energy metabolism. The detailed results are in Supplementary Table S9.

To further identify CRP’s potential pathways against LI, we also performed KEGG pathway enrichment analysis on 177 consensus genes, and the screen identified 49 pathways. For multiple pathways obtained by enrichment according to p. adjust (*p* < 0.01), we selected the top 20 paths for presentation (Figure 3G). The details of all KEGG pathway enrichment analyses are provided in Supplementary Table S10. Among these pathways, the interleukin (IL)-17 signaling pathway (hsa04657), endocrine resistance (hsa01522), the T cell receptor signaling pathway (hsa04660), Th1 and Th2 cell differentiation (hsa04658), platinum drug resistance (hsa01524), and others are critical pathways by which CRP acts on LI. Some of these are inflammatory pathways, some are apoptotic differentiation pathways, and others are receptor resistance pathways. Activation of inflammatory pathways such as IL-17 signaling (hsa04657) and tumor necrosis factor signaling (hsa04668) can significantly affect hepatocyte function (Yin and Feng, 2017; Doğanyiğit et al., 2020). Normal hepatocytes accelerate apoptosis after liver damage (Zhang T. et al., 2020). When CRP is administered to humans, the apoptosis pathway (hsa04210) may be affected by proteins such as BAX, BCL2, and CASP3 to maintain normal function. Liver function is also affected by the endocrine system, as endocrine resistance causes toxin-associated steatohepatitis (Clair, et al,2018).

### Computational Validation of Ingredients-Targets Interactions

According to the GO and KEGG results, we found that CRP anti-LI is a biological process with multiple targets and pathways. Hesperidin is highly abundant in CRP and is a major active component (Xia et al., 2006). Nobiletin has suitable ADME parameters, suggesting that it may exhibit good drug performance during LI treatment. Based on PPI analysis, we know that some genes linked to naringenin have high metrics. We believe that components such as hesperidin, nobiletin, and naringenin simultaneously affect LI through common targets. Therefore, these compounds were subjected to molecular docking with apoptosis regulators BAX, CASP3, and BCL2 to test our hypothesis. The lower binding energy between molecules represents more potent force binding energy between them, and <0 kcal/mol favors the binding reaction (Shi et al., 2020).

**FIGURE 4:**
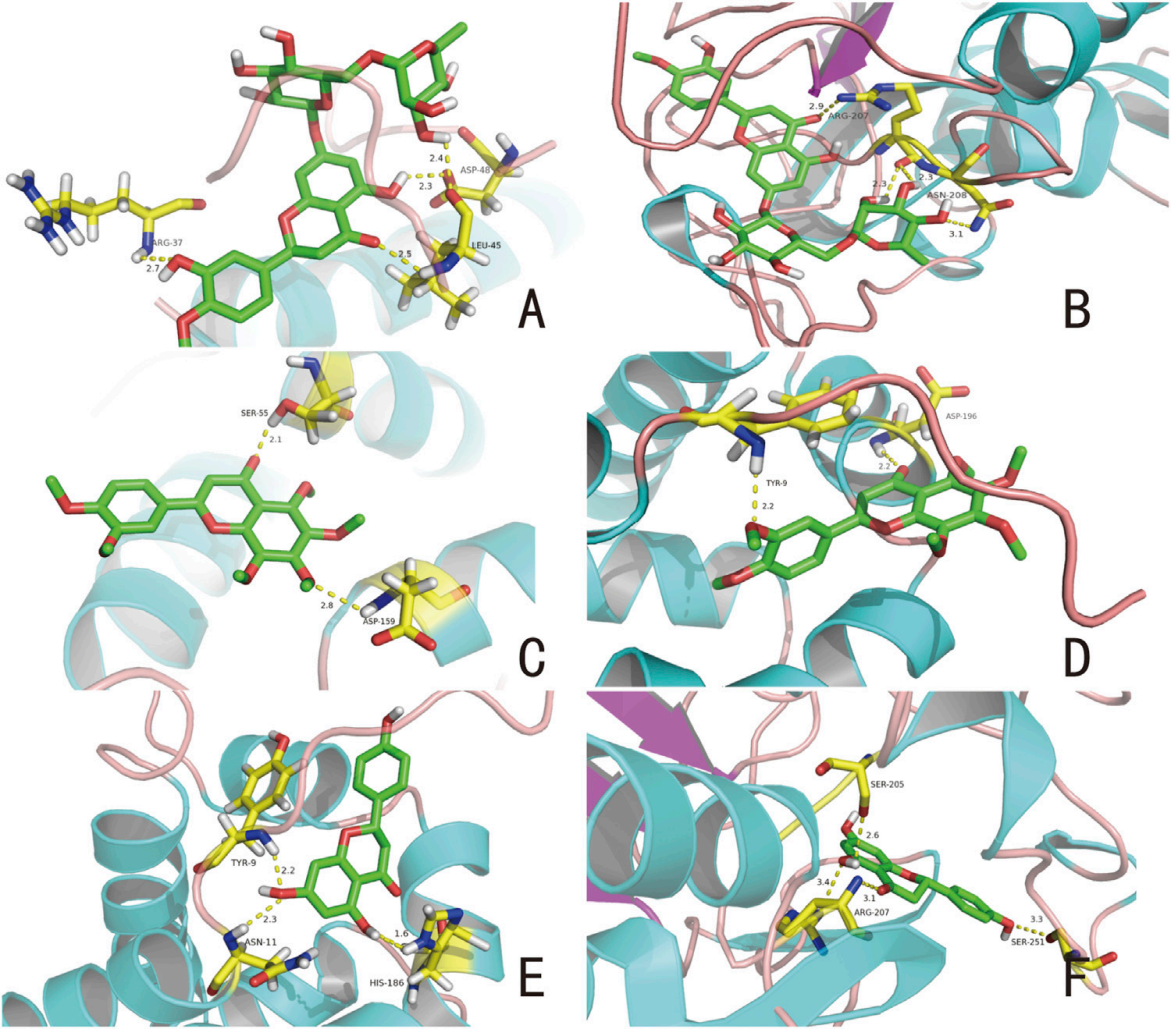
Selected compounds’ interactions with the targets. **(A)** Hesperidin with BAX, **(B)** hesperidin with CASP3, **(C)** nobiletin with BAX, **(D)** nobiletin with BCL2, **(E)** naringenin with BCL2, **(F)** naringenin with CASP3. The molecule is represented in a ball-stick model with atoms C, O, and N in green, red, and blue, respectively. Dashed lines indicate hydrogen bonds, and the numbers above represent distances in angstroms (Å).

According to the docking results, the binding energies of hesperidin to BAX and CASP3 were −8.2 and −8.6 kcal/mol, respectively; the binding energies of nobiletin to BAX and BCL2 were −5.9 and −7.7 kcal/mol, respectively; and the binding energies of naringenin to BCL2 and CASP3 were −7.7 and −7.2 kcal/mol, respectively. These results suggest that these compounds and their corresponding proteins have good binding. Figure 4 shows the combinations. Multiple linkages were formed between hesperidin and three amino acid residues in BAX, ARG-37, LEU-45, and ASP-48, including a C-O double bond and strong binding of the amino group (Figure 4A). Hesperidin interacts with two amino acid residues ASN-208 and ARG-207, in CASP3 (Figure 4B). These results suggest that a compound simultaneously forms multiple bonds with different amino acids in a protein, allowing the drug to affect the protein. The molecular docking results for nobiletin with BAX and BCL2 are shown in Figures 4C,D, respectively. The C-O double bond on nobiletin binds to the -OH group on SER-55 and the other O atom is connected to the H atom on ASP-519 (Figure 4C). The two O atoms on nobiletin and the amino acid residue TYR-9 on BCL2 and the H atom on ASP-196 form two hydrogen bonds (Figure 4D). Figures 4E,F show the docking results of naringenin with BCL2 and CASP3, respectively. The naringenin molecule binds to three residues (ASN-11, TYR-9, and HIS-186) on BCL2 to form multiple hydrogen bonds (Figure 4E). Naringenin also forms strong hydrophobic binding with residues ARG-207, SER-205, and SER-251 in CASP3 (Figure 4F). Our analysis showed that hesperidin, nobiletin, and naringenin effectively acts on protein targets closely related to apoptotic effects.

### Experimental Validation

#### Cell Viability

**FIGURE 5:**
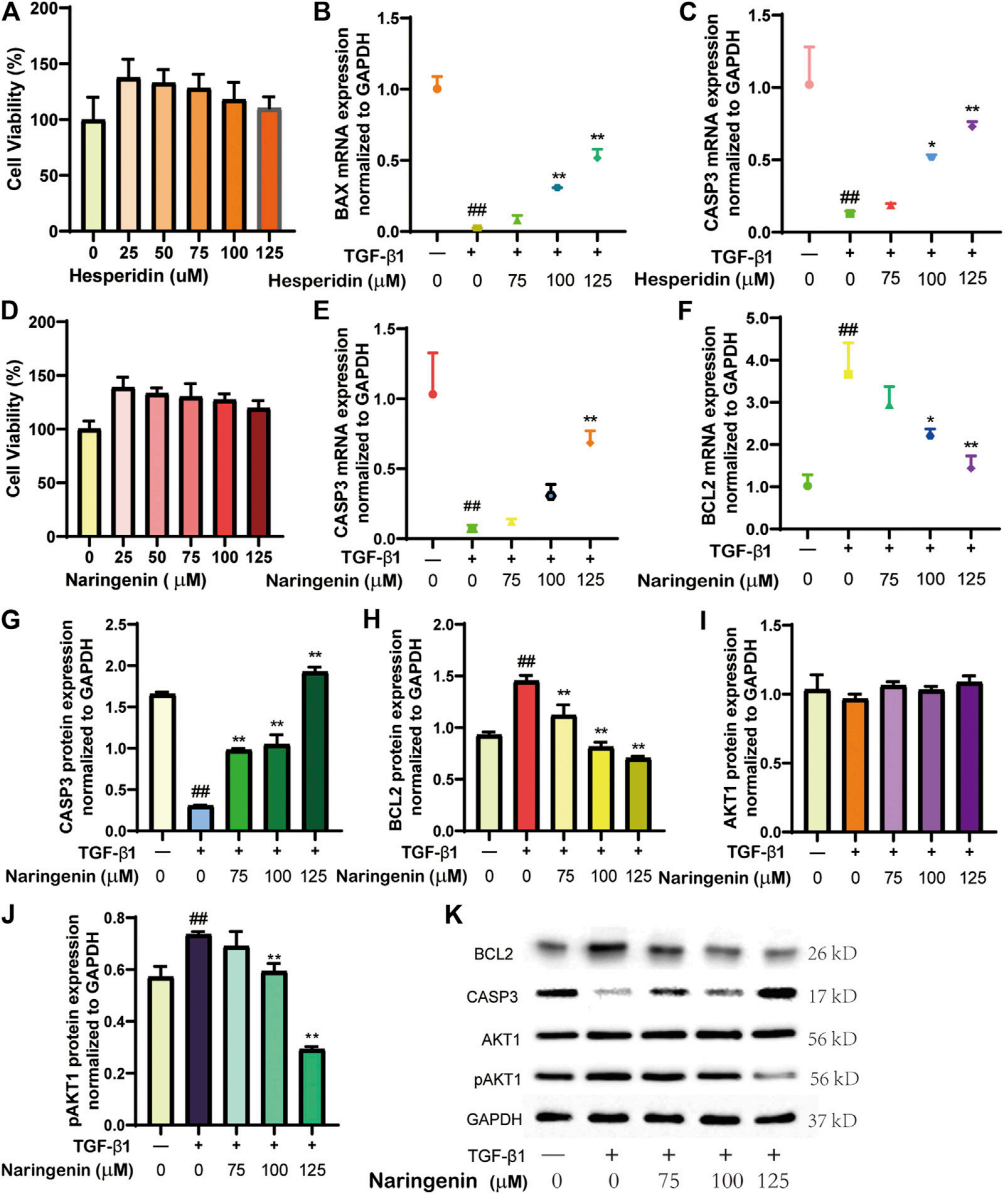
Effect of hesperidin or naringenin on LX-2 cells. The effects of hesperidin **(A)** or naringenin **(D)** on LX-2 cell viability using the CCK-8 assay. mRNA expression of BAX **(B)**, CASP3 **(C, E)**, and BCL2 **(F)** were determined by qRT-PCR. Protein levels of CASP3 **(G)**, BCL2 **(H)**, AKT1 **(I)**, pAKT1 **(J)** were determined by western blot, with results of each group **(K)**. ^#^ *p*< 0.05, ^##^ *p*< 0.01 versus blank control group; *p< 0.05, **p< 0.01 vs. TGF-β1-treated group.

CCK-8 assays were performed to determine the effects of different doses of hesperidin and naringenin on LX-2 cell viability (Figures 5A,D). When hesperidin or naringenin was added at concentrations >75 μmol/L, cell proliferation was not significant, and viability remained high (100–130%). Based on this, three concentrations (75, 100, and 125 μmol/L) were selected for subsequent experiments.

#### Target Validation

TGF-β1-induced activation of LX-2 cells predisposes to liver fibrosis, resulting from chronic LI developing into cirrhosis that ultimately leads to liver failure (Bestion et al., 2020; Xiang et al., 2020). We chose hesperidin and naringenin to evaluate our prediction results further to validate changes in several relevant proteins in LX-2 cells with or without TGF-β1 (5 ng/ml) treatment. We performed qRT-PCR to determine the effect of hesperidin on BAX and CASP3 mRNA levels and that of naringenin on CASP3 and BCL2 mRNA levels. Western blot was used to measure naringenin’s effect on CASP3, BCL2, ATK1, and pAKT1 protein levels. We found that mRNA levels of BAX and CASP3 were significantly lower after TGF-β1 treatment (*p* < 0.01, Figure 5B,C,E), BCL2 mRNA was significantly elevated (*p* < 0.01, Figure 5F), and mRNA levels of CASP3 and BCL2 were consistent with their protein expression results (Figures 5G,H). Compared with the group that only received TGF-β1 stimulation, The levels of multiple index proteins were adjusted to different extents after treatment with different concentrations of hesperidin or naringenin, which made the results obtained after compound treatment more similar to the normal group. Western blotting revealed that ATK1 was only weakly affected by TGF-β1 and naringenin; protein expression changes in each group were relatively stable (Figure 5I). Protein levels of pAKT1 increased rapidly after TGF-β1 treatment but decreased significantly after naringenin treatment (Figure 5J). This suggests that naringenin may have a more pronounced inhibitory effect on AKT1 phosphorylation. These data collectively suggest that CRP reduces LI by mediating cell apoptosis and protein phosphorylation and regulating BAX expression, CASP3, BCL2, and ATK1, all of which are consistent with our molecular docking results. The results support our predicted results based on systems pharmacology.

## Discussion

The liver is both a metabolic organ and an important mediator of immune function. LI may cause cancer, and the use of anti-cancer drugs may lead to more severe liver damage (Björnsson et al., 2020). TCM originates from nature and has few side effects, but its application and development have some limitations due to the complexity of the compositions and mechanisms of action. CRP is a pure, natural TCM that can also be used as a food, and a variety of compounds in CRP have been shown to have anti-proliferative effects on hepatoma cells (Chu et al., 2017).

In this study, we identified the active components and related targets of CRP. We linked them to LI-related targets to construct a D-C-G-D network that depicts links among 117 consensus genes and diseases. The PPI analysis showed complex interactions between 117 overlapping genes, and topological value analysis further indicated 25 core targets. We also examined these targets’ expression relationships in the liver, heart, lung, kidney, small intestine, and colon. The results showed that their expression levels were similar in these organs that belong to the TCM category of “five viscera and six entrails.” The theory of TCM posits that the viscera is an organic whole, the center of human life activities, and the organs work together to maintain the living body.

The GO analysis results revealed biological connections between drugs and diseases, and the BP enrichment clarified the response to the stimulation of external substances, such as response to acid chemical (GO:0001101). Hepatocytes are more sensitive to exogenous substance stimulation, and liver lesions develop when individuals regularly work in environments containing chemicals (Zhao et al., 2020). The CC analysis revealed the cellular environment in which the intersection protein or its product occurred, including membrane raft (GO: 0045121). Alcohol alters plasma membranes *in vitro*; preventing membrane raft oxidation is one approach to inhibit LI caused by excessive alcohol consumption (Chen et al., 2018). MF results suggest how intersection proteins or their products bind or function *in vivo* after the compound performs its action. Among them, catecholamine binding (GO: 1901338) is related to the expression of genes, and catecholamine receptors’ regulation prevents acute stress-induced LI (Zhu et al., 2014). We also found that a large number of GO terms were related to mitochondria, including regulation of mitochondrial membrane potential (hsa04210), apoptotic mitochondrial changes (GO: 0008637), and mitochondrial outer membrane permeabilization (GO: 0097345). This finding suggests that one of the critical mechanisms by which CRP ameliorates LI is to alter cellular energy metabolism (Xu et al., 2017; Dilberger et al., 2019).

The KEGG pathway enrichment results revealed that inflammatory and apoptotic pathways might be the primary CRP action pathways. CRP mediates the IL-17 signaling pathway by regulating targets such as PTGS2, MAPK3, and CASP3. PTGS2, also known as cyclooxygenase 2, extensively participates in hepatic inflammation induced by xenobiotics (Zhang et al., 2019). Through the D-C-G-D network, we know that hesperidin, nobiletin, and tangeretin are all involved in regulating PTGS2. The inhibition of PTGS2 mRNA expression is one of the manifestations of LI’s acute inflammation resolution (Li et al., 2020). It also suggests that a protein can be affected by several compounds simultaneously. LX-2 cells are prone to proliferate faster, and this can lead to liver fibrosis (Bestion et al., 2020). Our *in vitro* results suggest that CRP can induce apoptosis in LX-2 cells by regulating targets such as BAX, BCL2, and CASP3, consistent with our finding that the apoptosis pathway (hsa04210) is affected by CRP. The liver has a unique regenerative capacity (Nejak-Bowen and Monga, 2011). Some cancer pathways are associated with uncontrolled cell growth, and their dysregulation can lead to liver cancer. The phosphoinositide 3-kinase (PI3K)-Akt signaling pathway (hsa04151) can be activated by a variety of cell stimulants and toxins and is potentially correlated with liver cancer (Shi et al., 2020). Our prediction results suggest that AKT1 has a high degree in this pathway, and the phosphorylated form is a biomarker for cancer and tumor biology (Balasuriya et al., 2020). Our *in vitro* validation experiments demonstrated that naringenin significantly inhibited AKT1 phosphorylation, which in turn mediated PI3K-Akt signaling pathways to counteract LI. This interesting finding will be the focus of our future study. Nobiletin in CRP inhibits hepatoma metastasis resulting from PI3K-Akt signaling and may become a new compound for liver cancer treatment (Shi et al., 2013). The western blot results indicated that naringenin significantly reduced pAKT1 levels, suggesting that it prevents LI exacerbation. Increased cytochrome P450 expression improves hepatocyte function (Lewis et al., 2020); xenobiotics’ metabolism by cytochrome P450 (HSA00980) pathway was analyzed. We found that it resulted from naringenin, tangeretin, narirutin, and other compounds by affecting GSTP1, CYP1A1, and CBR1 targets.

We propose the following mechanism by which CRP counteracts LI. Hesperidin and naringenin in CRP affect the apoptosis pathway by acting on CASP3, BAX, and BCL2, reducing the likelihood of liver fibrosis. Naringenin significantly inhibits AKT1 phosphorylation, in turn mediating PI3K-Akt signaling to counteract LI. Systems pharmacology is a useful strategy to predict therapeutic mechanisms and identify new research directions. We acknowledge that the predictions and actual situations may be biased; however, they complement the *in vitro* findings, consistent with the molecular docking results that test the system pharmacology-based screening strategy’s reliability from a different perspective.

## Conclusion

Previous drug development models mainly followed the concept of “one drug, one gene, and one disease.” In this study, modern technologies such as UPLC-Q-TOF-MS/MS and molecular docking were used to clarify the specific modes of the synergistic effects of multiple CRP components against LI, and provided convincing evidence. System pharmacology of TCM is an emerging multi-field interdisciplinary, but its development still has certain limitations. In addition to being an important reference for CRP in the treatment of liver diseases, our research also has many shortcomings. The specific interactions between all the drug components, proteins and multiple signal pathways involved in it need to be further studied. This approach of Systems pharmacology is a powerful new way to elucidate the mystery of TCM.

## Data Availability

The original contributions presented in the study are included in the article/Supplementary Material, further inquiries can be directed to the corresponding authors.
